# Instability of some equatorially trapped waves

**DOI:** 10.1002/jgrc.20219

**Published:** 2013-06-10

**Authors:** Adrian Constantin, Pierre Germain

**Affiliations:** 1 Department of Mathematics, King’s College LondonStrand, Westminster, UK; 2 Faculty of Mathematics, University of ViennaNordbergstrasse, Vienna, Austria; 3 Courant Institute of Mathematical Sciences, New York UniversityNew York, USA

**Keywords:** equatorially trapped wave, instability

## Abstract

[1] A high-frequency asymptotics approach within the Lagrangian framework shows that some exact equatorially trapped three-dimensional waves are linearly unstable when their steepness exceeds a specific threshold.

**Citation:** Constantin, A., and P. Germain (2013), Instability of some equatorially trapped waves, J. Geophys. Res. Oceans, 118, 2802–2810, doi:10.1002/jgrc.20219.

## 1. Introduction

[2] Exact solutions play an important role in the study of geophysical flows since many apparently intangible wave motions can often be viewed as perturbations thereof. By controlling the perturbations one can extract relevant information (qualitative as well as quantitative) about the dynamics of more complex flows. The successful implementation of this approach is contingent upon two aspects. First, it is essential to unveil as much as possible the detailed structure of the exact solution. Explicit solutions for ideal flows are known in the Lagrangian and in the Eulerian framework but only the simplest ones have a tractable form in both descriptions. Since in the Eulerian formalism the flow is described by the determination of the fluid velocity at fixed points in space as a function of time, whereas in the Lagrangian framework one specifies the motion of the individual particles, Lagrangian solutions present the great advantage that the fluid kinematics may be described explicitly [see, *Bennett*, 2006]. Once an exact solution is available, the stability issue becomes important. For stable flows small perturbations do not alter the main characteristics of the motion—all perturbations which are small initially remain small for all time. Instability occurs when the effect of some disturbance of the forces acting on the fluid grows as time progresses. The nature of the instability is important for understanding the factors that might trigger the transition from the large-scale coherent structure represented by the exact solution to a more chaotic fluid motion.

[3] The aim of the paper is to present a stability analysis of the three-dimensional geophysical flow that was recently derived by *Constantin* [2012]. The explicit Lagrangian solution to the governing equations in the *β*-plane approximation describes the eastward propagation of equatorially trapped waves (see section 2), having only an implicit representation within the Eulerian description of fluid flows. The investigation of the stability of this wave pattern relies on the implementation of the theory of short-wavelength instabilities developed independently by *Bayly* [1987], *Friedlander and Vishik* [1991], and *Lifschitz and Hameiri* [1991] (see also the survey, *Friedlander and Yudovich* [1999]). In section 3.1, we present the short-wavelength instability approach for geophysical equatorial flows, while section 3.2 is devoted to applying the method to the specific study of the equatorially trapped waves. In particular, we identify perturbations in the meridional direction as a source of instabilities with an exponentially growing amplitude and we show that the growth rate of the instabilities depends on the steepness of the wave profile travelling eastward along the Equator.

## 2. Description of the Equatorially Trapped Wave

[4] Explicit solutions for gravity fluid flow within the Lagrangian framework, while very desirable from a kinematical viewpoint, are not that numerous. Moreover, those solutions describing flows with a free surface are all generalizations of the rotational deep water gravity water wave due to *Gerstner* [1809]–see, the discussions in *Aleman and Constantin* [2012]; *Constantin* [2001]; *Stuhlmeier* [2011]; *Weber* [2011]. Recently, the approach pioneered by Gerstner was extended by *Constantin* [2012] to geophysical fluid flows. We now present the main features of this specific explicit equatorially trapped solution.

**Figure 1:**
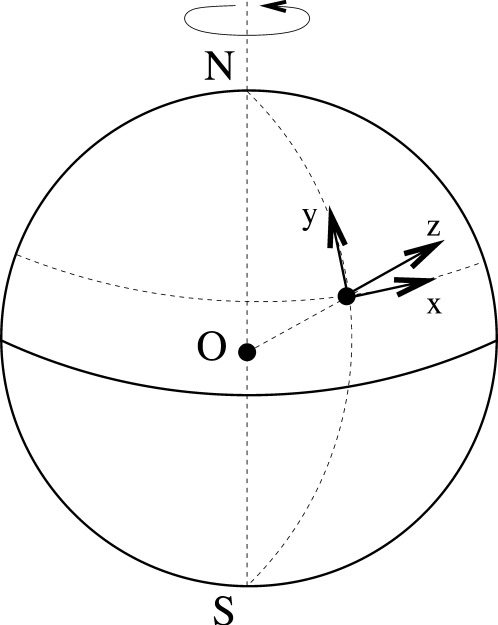
The rotating frame of reference with the origin at a point on the Earth’s surface: *x* corresponds to longitude, *y* to latitude, and *z* to the local vertical.

[5] The geophysical wave is symmetric about the Equator, being confined to a region within 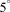 latitude from the Equator (corresponding to an equatorial band of width about 1110 km, centered on the Equator). Approximating the shape of the Earth by a sphere of radius *R* = 6378 km, consider a rotating framework with the origin at a point on the Earth’s surface, with the *x* axis chosen horizontally due east, the *y* axis horizontally due north and the *z* axis upward (see Figure 1). Since the meridional distance from the Equator is moderate, the governing equations for geophysical ocean waves are the Euler equations in the *β*-plane approximation,


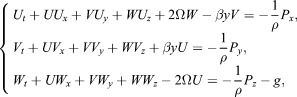
(1)

together with the condition of incompressibility


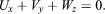
(2)

[6] On the free surface the kinematic boundary condition


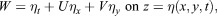
(3)

for an impermeable free surface–a material boundary across which there is no flux of matter of the macroscopic scale (a particle initially on the boundary will remain on the boundary), and the dynamic boundary condition


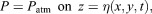
(4)

requiring the stress to be continuous across the free surface which separates the air and the water, must hold. In (4) *P*_atm_ stands for the (constant) atmospheric pressure, thus decoupling the water flow from the motion of the air above it (see, the discussion in *Constantin* [2011]). We also require the velocity field to decay rapidly with depth, so that at great depth there is practically no motion. In (1) and (2), 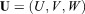 is the fluid velocity, 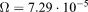 rad 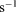 is the (constant) rotational speed of the Earth round the polar axis toward the east, 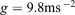 is the (constant) gravitational acceleration at the Earth’s surface, *ρ* is the constant water’s density, and *P* is the pressure. The *β*-plane effect 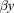 noticeable in (1), with 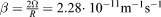, is the result of linearizing the Coriolis force in the tangent plane approximation: although the Earth was assumed to be spherical, since the spatial scale of motion is moderate, the region occupied by the fluid can be approximated by a tangent plane and the linear term of the Taylor expansion captures the *β*-plane effect [see, the discussions in *Cushman-Roisin and Beckers*, 2011; *Gallagher and Saint-Raymond*, 2007].

[7] Let the Lagrangian positions 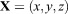 of fluid particles be given as functions of the labeling variables 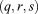 and time *t* by


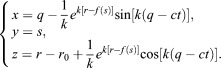
(5)

[8] Here *k* is the wave number,


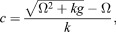
(6)

is the wave speed, and


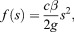
(7)

captures the decay of particle oscillation in the meridional direction. The labeling variables *q* and *s* cover the real line, while 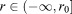 for some fixed 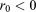. Here 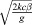 can be identified as the inverse equatorial Rossby radius 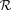 for this problem. Since 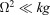 for all physically reasonable waves, 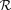 can be approximated by 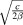, which is the form given by *Gill and Clarke* [1974] in the barotropic shallow-water case.

**Figure 2:**
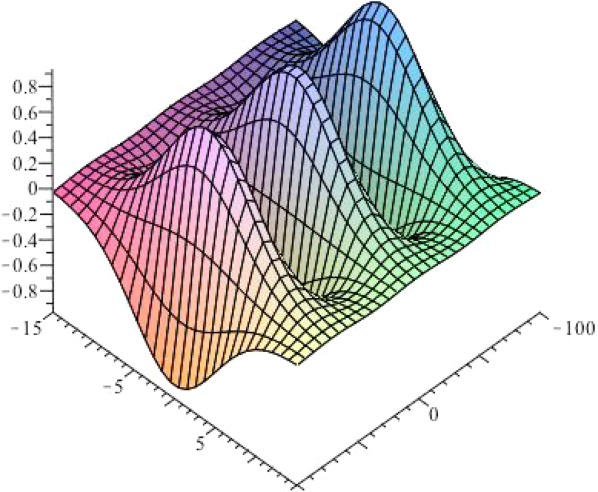
Depiction of an equatorially trapped eastward propagating surface wave. The maximal amplitude is attained along the Equator. [One unit in the vertical scale stands for 4 m, the 100 longitudinal units stand for 200 m, and the 30 meridional units stand for 1000 km.]

**Figure 3:**
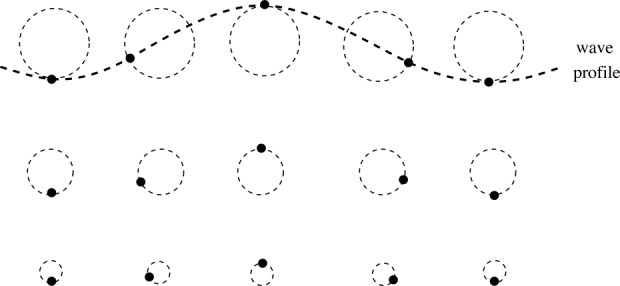
The motion at a fixed latitude: each particle beneath the surface wave describes a circle, and they are all in the same phase. The diameter of these circles decreases exponentially with the distance to the surface. In particular, at a depth of half a wavelength the diameter is reduced to about 4% of its free-surface value, illustrating the deep water wave character of the motion.

**Figure 4:**
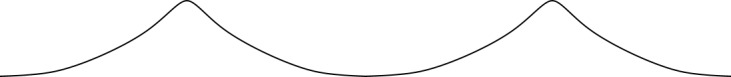
The surface wave profile at a fixed latitude is an inverted trochoid. Its sharp, narrow crests and relatively flat troughs contrast with the features presented by the sinusoidal waves encountered in linear theory. As the wave steepness increases, the departure from a sinusoidal profile is more salient. [The steepness of these profiles does not exceed 1 and the crest and trough are not equidistant from the mean level.]

[9] The equation (5) define an equatorially trapped wave propagating eastward, the free surface 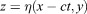 at latitude *y* = *s* being obtained by setting 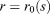, where 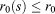 is the unique solution to 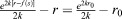. The fluid velocity field, the pressure and the free surface exhibit an (*x,t*) dependence of the form (*x–ct*), the flow is oriented eastward with a vanishing meridional velocity *V*, and all particles move in a vertical plane. Note that all particles move on circles (see Figure 2), a feature that is in contrast to the case of irrotational gravity water waves [discussed in *Constantin*, 2006; *Constantin and Strauss*, 2010; *Henry*, 2008]. The restriction of the flow pattern to a fixed latitude replicates a two-dimensional wave motion (see Figure 3), with the three-dimensional character of the flow captured by the decay in the meridional direction. The considerations in *Constantin* [2012] show that the explicit tractable form (5) of the flow in Lagrangian coordinates corresponds to an intricate implicit representation of the velocity field within the Eulerian framework. This feature is highlighted by the fact that at each fixed latitude *y* = *s*, the free surface profile 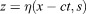 is trochoidal (see Figure 4). We emphasize the nonlinear character of the flow pattern (5). The particle velocity can be computed from (5) as


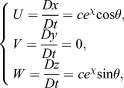
(8)

where we set


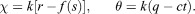


[10] Furthermore [see *Constantin*, 2012]


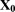


while the vorticity 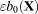 of the flow is


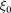


[11] Consequently, in the Euler equation (1) with 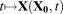, both terms 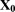 and 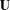 have orders of magnitude in a ratio 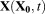 since 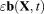 are all of order 1 due to constraint 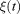. Note that along the Equator we have 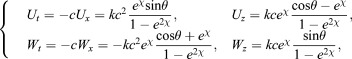, so that by (5) the steepness of the equatorial wave profile, defined as the amplitude multiplied by the wave number, is precisely 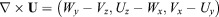 since the height (that is, the difference in elevation between the crest and the trough) of the wave propagating eastward along the Equator is 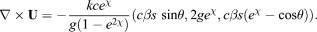. The above considerations show that an increasing steepness enhances the nonlinear character of the flow (5). A further issue of interest is the significance of the Coriolis terms 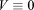. For the wavelength *L* = 63 m we have 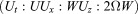, so that (6) yields 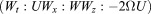. Choosing 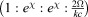, depths of about 53 m beneath the surface correspond to 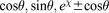. This yields 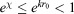. Therefore at these specific depths along the Equator, 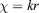, being roughly 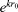, is comparable to 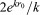, thus confirming the geophysical character of the flow. Note that while at depths in excess of 88 m beneath the surface the water is practically still since for 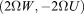 the height of the particle oscillation (5) is of the order 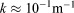, at about 50 m beneath the surface the motion induced by the surface wave is not negligible since the height of the particle oscillation (5) is of the order 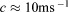. Also, since the wave steepness of the wave profile propagating along the Equator is 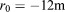, the linear and the weakly nonlinear regime are not appropriate and the wave motion is genuinely nonlinear. For this concrete example the maximal wave height of the trapped wave is 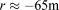, so that the wave can be classified among the relatively high waves [cf., *Holden*, 2012; *Kinsman*, 1965]. The corresponding equatorial radius of deformation 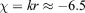 is approximately 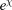. At meridional distances 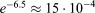 and 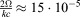 from the Equator the wave height reduces to 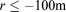 and 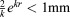 respectively. Note that for this concrete example the vorticity 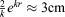 of the flow in a near-surface layer 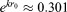 centred on the Equator, less than 88 m deep, extending about 1000 km in the meridional direction and all across the whole length (about 13,000 km) of the ocean basin, is well approximated by 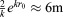. Since at depths in excess of 88 m beneath the surface the motion is practically ground to a halt, the above formula for the vorticity is indicative of a near-surface westward flowing current. To elucidate this aspect, let us fix the latitude *s*. Since by (8) the average of the horizontal fluid velocity *U* over a wave period 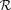 clearly vanishes, the mean Lagrangian velocity is zero:


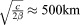


[12] Note that the crest/trough levels of the surface wave that propagates eastward at latitude *s* are 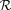, while the mean water level is


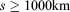


as one can see by using in the second step the outcome of the differentiation of the *x* component of (5) with respect to the *q* variable. A fixed depth 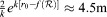 beneath the level 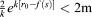 of the surface wave troughs is characterized in Lagrangian variables by means of the relation


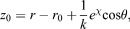
(9)

which yields a functional dependence


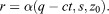
(10)

[13] By differentiating (9) with respect to the *q* variable we obtain


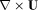


so that


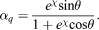
(11)

[14] This permits us to compute the Eulerian mean velocity 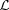 at the latitude *s* and at the depth 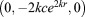 from


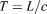


due to (5), (8), (10), (11). Thus


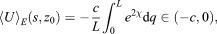
(12)

which shows that the Eulerian mean flow is westward. Since the Lagrangian mean flow is zero, the Stokes drift, defined by *Longuet-Higgins* [1969], as the difference between the Lagrangian and the Eulerian mean velocities, is eastward. Note that differentiating (9) with respect to *z*_0_ yields


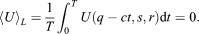


so that


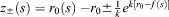


[15] Combining this with (12), we get


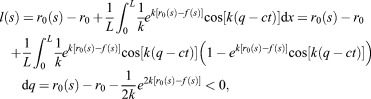


[16] Therefore the Eulerian mean velocity 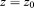 depends monotonically on the depth, with the westward flow of significance only in the near-surface region and barely noticeable at great depths. This is the hallmark of a nonuniform wave-induced westward current.

[17] We conclude our discussion of the flow (5) by investigating the mass transport. Recall that 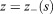 is sometimes called the mass-transport velocity, being the mean velocity of a marked particle [cf., *Longuet-Higgins*, 1969; *Constantin*, 2013]. Let us compute the mass flux past 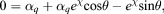 at a fixed latitude *s*, given by


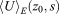


and representing the instantaneous zonal transport across a fixed longitude. From (8) and differentiating with respect to *r* the third component of (5), evaluated at 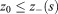 determined by the constraint


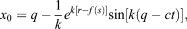
(13)

we get


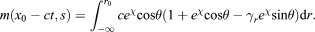
(14)

[18] Differentiating (13) with respect to *r* yields


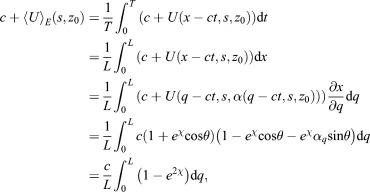


so that


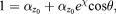


and (14) becomes


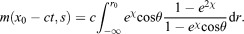
(15)

[19] Note that the wave crests/troughs lie on the line 
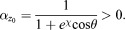
 if and only if 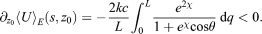, in which case (15) shows that mass is carried forward/backward, respectively. Since 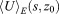, the average of the mass flux over a wave period *T* vanishes. This fact is encoded in (15): differentiating (13) with respect to *t* yields


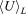


so that


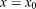


and (15) takes on the form


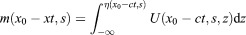


[20] The *T*-periodicity of the function 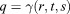 confirms that the average of the mass flux over a period *T* vanishes.

## 3. Instability Analysis

[21] In this section, we first present the short-wavelength instability approach for a general geophysical equatorial flow. Subsequently, we study the specific case of the equatorially trapped waves.

### 3.1. Short-Wavelength Instability Approach

**Figure 5:**
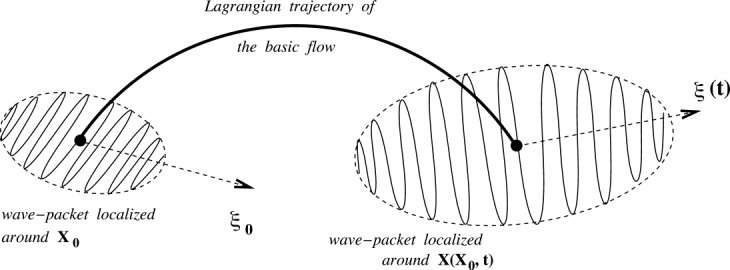
The initial high-frequency disturbance, localized near 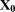, is moved by the flow. Both its amplitude 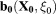 and its normalized wave vector 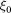 change throughout this process. If 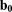 is the Lagrangian trajectory of the particle with label 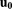, transported by the basic flow 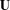, at a later time *t* the disturbance evolves to a wave packet localized near 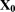, of amplitude 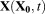 and with normalized wave vector 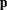.

[22] Since the stability issue concerns the evolution of small perturbations with time [cf., *Drazin*, 2002; *Yudovich*, 1984], it is reasonable to pursue its investigation within a linear framework by neglecting nonlinear terms arising from products of the perturbed quantities. The stability analysis of a basic flow represented by the velocity field 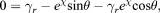 of an inviscid incompressible fluid relies on the study of the growth of infinitesimal disturbances 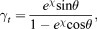. Within the short-wavelength instability approach one considers the evolution of a rapidly varying localized wave packet following a particle. Choose an initial disturbance in the form


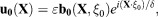
(16)

where the vector 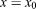 represents the normalized amplitude, 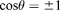 is the normalized wave vector subject to the transversality condition 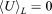, and the small parameters *ε* and *δ* ensure that the small disturbance oscillates rapidly in space (see Figure 5). The choice of a function 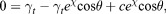 of small support and sharply peaked at a point 
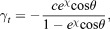
 in the fluid localizes the initial disturbance near 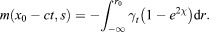 and specifies 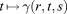 as its main direction. The orthogonality between the wave vector 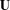 and the wave amplitude 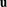 is forced by the incompressibility constraint (see later) and confers to the perturbation the characteristic of transverse waves, thus inducing a particle displacement that is orthogonal to the direction of wave propagation. The initial disturbance 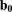 is moved by the basic flow 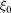, so that at time *t* the fluid particle at 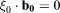 has moved to a point 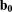. With this specific choice of initial disturbance, we represent, at leading order in powers of *ε*, the subsequent evolution of the velocity and pressure perturbations in the form


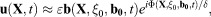
(17)

and


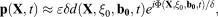
(18)

respectively, where the scalar function *d* measures the amplitude of the pressure perturbation 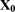. The initial conditions (at *t* = 0) are


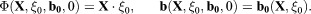
(19)

[23] Writing (1) and (2) for the perturbed flow leads to


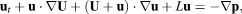
(20)

and


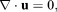
(21)

respectively, where we denoted


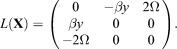
(22)

[24] At highest order in the expansion of (21) in powers of *δ*, due to (17), we have


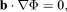
(23)

while, as long as 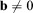, (20) yields


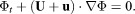
(24)

[25] Since (23) is equivalent to 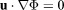, the above equation leads to the eikonal equation


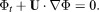
(25)

[26] Taking the gradient of (25) gives now the evolution equation


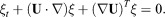
(26)

for the field of wave vectors 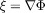, where 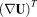 is the transpose of the basic velocity gradient tensor 
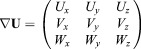
.

[27] On the other hand, at the highest order in the expansion of (20) in powers of *ε*, we have


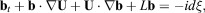
(27)

if we recall (24). Taking the time derivative of (23), and taking advantage of (26) and (27) leads to


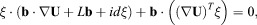


since 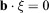 in view of (23). Solving for *d*, we obtain


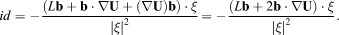


[28] The above expression can now be used in (27) to yield


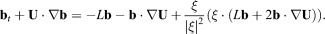
(28)

[29] At leading order in powers of *ε*, the path of the particle located initially at 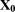 is determined by solving the ordinary differential equation 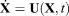 with initial data 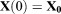 : the perturbation velocity field 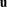 does not contribute to the advection of the observation point 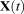. Since the material derivative 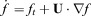 describes the rate of the change of the vector *f* as a particle moves due to the bulk flow (induced by the unperturbed velocity field 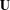 ), taking into account (26) and (28), we deduce that the evolution in time of 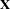, of the amplitude 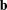 and of the wave vector 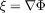 is governed, at leading order in an expansion in powers of *ε* and *δ*, by the specific coupled system of ordinary differential equations


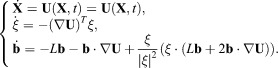
(29)

[30] The initial conditions (at *s* = 0) associated to (29) are


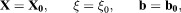
(30)

with 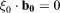. The growth rate of the vector 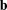 is analogous to the concept of a Lyapunov exponent for a dynamical system: if for some initial position 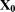 we have


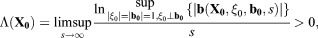


then exponential stretching occurs in the flow – certain particles are separated at an exponential rate and this separation, visualized as stretching, is a hallmark of instability [cf., the discussion in *Friedlander*, 1999; *Friedlander and Yudovich*, 1999]. From the point of view of spectral analysis, if 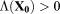 for some 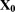, then the unbounded operator associated to the linearization around the basic flow has a nonempty unstable continuous spectrum in the space of square integrable functions [cf., *Friedlander and Yudovich*, 1999]. Note that while in (29) the second and third equation are linear, the first equation is usually nonlinear but decouples from the other two. The first equation provides the particle trajectory of the basic (undisturbed) flow, while the second and third equation govern to leading order the evolution along this trajectory of the local wave vector and of the amplitude of the perturbation, respectively.

### 3.2. Instability of the Equatorially Trapped Wave

[31] To prove the instability of the geophysical fluid flow (5), it is not necessary to investigate the associated system (29) for all initial data. It suffices to exhibit a choice for the initial disturbance that results in an exponentially growing amplitude 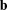. This is our aim.

[32] The class of disturbances characterized by 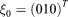 is of particular interest since in this case the solution to the equation for *ξ* in (29) can be computed explicitly: 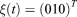 for all 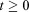. Using this fact, we see that the equation for 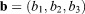 in (29) becomes


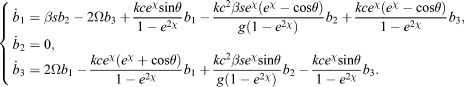
(31)

[33] The dependence of *θ* on *t* prevents the above linear differential system from being autonomous. Thus the possibility of solving (31) explicitly seems remote. Fortunately, there is some hidden structure of the system. First of all, the choice 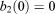 ensures 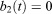 for all 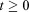, so that 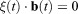 for all 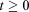. Moreover, (31) is reduced to the planar nonautonomous linear differential equation


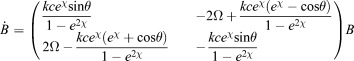
(32)

for 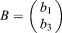. Write (32) in the form


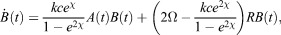


with


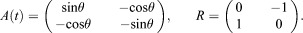


[34] In the rotating frame of reference corresponding to the change of variables induced by the matrix


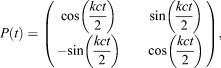


the system (32) becomes autonomous and therefore tractable. Indeed,


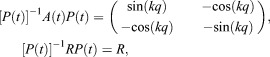


while


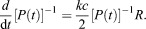


[35] Therefore, if we set 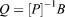, then


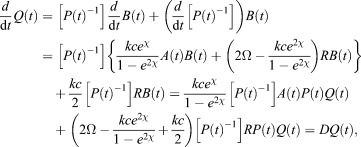


where


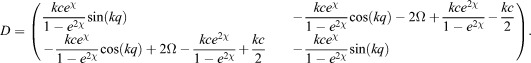


[36] The asymptotic behaviour of *Q*(*t*) as 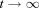 is determined by the eigenvalues of the matrix *D*. These are found by solving the quadratic equation


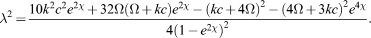
(33)

[37] Consequently, exponential growth of the solution *Q*(*t*) occurs for 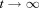 if and only if


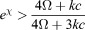
(34)

(recall from section 2 that 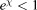 ). Using (6), we can express (34) as


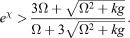
(35)

[38] Since the matrix *P*(*t*) is time periodic and 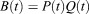, the temporal growth of the short-wavelength disturbances 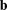 is exponential if the wave propagating eastward along the Equator is sufficiently steep. Indeed, recall from section 2 that the steepness of the equatorial wave profile is precisely 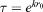. The exponential growth rate of the short-wavelength disturbances is given by the positive root of the equation (33). These considerations shows that the equatorially trapped wave (5) with wave number *k* is linearly unstable when the steepness *τ* of the wave propagating eastward along the Equator is strictly larger than the expression on the right-hand side of (35)–(35): an initial disturbance of the form 
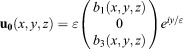
, localized in the near-surface layer, will grow exponentially fast at the rate


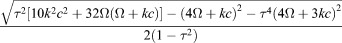
(36)

given by the positive root of equation (33). For all physically realistic equatorially trapped waves we have that 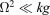. Consequently, while the right-hand side of (35) is always larger than 1/3, it is very close to 1/3. The previous considerations show that a steepness of the wave propagating eastward along the Equator in excess of 1/3 triggers instability.

## 4. Discussion

[39] The theory of short-wavelength perturbations has been applied to prove the linear instability of some recently derived equatorially trapped waves when the wave profile that propagates along the Equator is sufficiently steep. Despite the efficiency of the approach for steep waves, beneath the threshold specified in (34) and (35) this method appears to be inconclusive. Even in this regime stability is unlikely but analytic or numerical evidence is currently not available.

[40] By inspection one can see that the instability condition (34) simplifies to the requirement that the wave steepness exceeds 1/3 if one eliminates the small 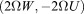 terms in the equation (1). In particular, setting 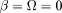 in (5), we obtain Gerstner’s gravity water wave and we recover the results obtained by *Leblanc* [2004], investigation that appears to be the first stability analysis of a Lagrangian flow that is not explicitly available in the Eulerian representation and was a source of inspiration for the present paper.

[41] It is of interest to compare our results with the studies of the effect of zonal currents on surface equatorial Kelvin waves – waves that are usually associated with anomalies in surface wind stress and penetrate the entire depth of the ocean [cf., *McPhaden and Ripa*, 1990; *Wang*, 2003]. Like (5), these equatorially trapped waves propagate eastward and their meridional velocity vanishes everywhere. However, unlike (5), the equatorial Kelvin waves are not exact solutions to the governing equations (1)-(4) but approximate linear solutions in the shallow water regime: neglecting the vertical motion and with a vanishing meridional velocity, the linearized shallow-water equations in the equatorial *β*-plane take the form


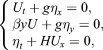
(37)

[42] [cf., *Gill*, 1982]. In (37) the constant *H* is the mean depth of the fluid and *η* is the perturbation of the flat free surface *z* = 0. Note that the first and third equation in (37) yield the linear wave equation 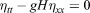. Seeking solutions in the form 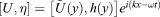, the dispersion relation 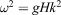, relating the frequency *ω* and the wave number *k*, emerges. The third equation in (37) forces 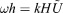, so that the second equation in (37) becomes


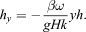
(38)

[43] Since the solution with exponential growth has to be ruled out on physical grounds, the only realistic solution is the equatorial Kelvin wave


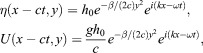


propagating eastward with speed 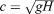. The surface equatorial Kelvin waves are destabilized by a weak cross-equatorial shear of the type 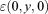, modeling a mean equatorial current with meridional shear: numerical studies [see, *Boyd and Christidis*, 1982; *Boyd and Natarov*, 2002; *Boyd*, 2005; *Natarov and Boyd*, 2001] show that the imaginary part of the complex phase speed (i.e., the growth rate) is an exponentially small function of the strength *ε* of the cross-equatorial shear, being of order 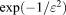. In contrast to this, the instability mechanism for steep equatorially trapped waves of type (5) predicts an exponential growth rate of the instabilities that depends on the steepness of the wave profile traveling eastward along the Equator.
